# Antibacterial Porous Systems Based on Polylactide Loaded with Amikacin

**DOI:** 10.3390/molecules27207045

**Published:** 2022-10-19

**Authors:** Marta Glinka, Katerina Filatova, Justyna Kucińska-Lipka, Tomáš Šopík, Eva Domincová Bergerová, Veronika Mikulcová, Andrzej Wasik, Vladimir Sedlařík

**Affiliations:** 1 Department of Analytical Chemistry, Faculty of Chemistry, Gdańsk University of Technology, 11/12 G. Narutowicza Street, 80-233 Gdańsk, Poland; 2 Centre of Polymer Systems, University Institute, Tomas Bata University in Zlín, Tomáše Bati 5678 Street, 760 01 Zlín, Czech Republic; 3 Department of Polymer Technology, Faculty of Chemistry, Gdańsk University of Technology, 11/12 G. Narutowicza Street, 80-233 Gdańsk, Poland

**Keywords:** poly(lactic acid), amikacin, drug delivery systems, porous matrices, tissue engineering

## Abstract

Three porous matrices based on poly(lactic acid) are proposed herein for the controlled release of amikacin. The materials were fabricated by the method of spraying a surface liquid. Description is given as to the possibility of employing a modifier, such as a silica nanocarrier, for prolonging the release of amikacin, in addition to using chitosan to improve the properties of the materials, e.g., stability and sorption capacity. Depending on their actual composition, the materials exhibited varied efficacy for drug loading, as follows: 25.4 ± 2.2 μg/mg (matrices with 0.05% *w*/*v* of chitosan), 93 ± 13 μg/mg (with 0.08% *w*/*v* SiO_2_ amikacin modified nanoparticles), and 96 ± 34 μg/mg (matrices without functional additives). An in vitro study confirmed extended release of the drug (amikacin, over 60 days), carried out in accordance with the mathematical Kosmyer–Pepas model for all the materials tested. The matrices were also evaluated for their effectiveness in inhibiting the growth of bacteria such as *Staphylococcus aureus*, *Escherichia coli*, *Klebsiella pneumoniae*, and *Pseudomonas aeruginosa*. Concurrent research was conducted on the transdermal absorption, morphology, elemental composition, and thermogravimetric properties of the released drug.

## 1. Introduction

Bacterial infections under wound dressings pose the most common problem in the treatment of wounds and burns, resulting in scarring, sepsis, and even death. Drawbacks inherent to traditional materials for dressing wounds, such as undesirable adhesion to the wound and insufficient water and air permeability, mean they often do not meet the criteria for proper and satisfactory healing. As a consequence, a wide range of polymer-based systems have been investigated over the past decade [1,2,3,4,5].

It is possible to fabricate polymer-based systems for wound dressings from natural or synthetic polymers. The former of the two, e.g., proteins and polysaccharides, excel for the biocompatibility they exhibit [2,6]. Commercial use could prove complex, though, for reasons of the cost of natural polymers and the potential for subtle changes in properties depending on their origin. This is why synthetic polymers are usually preferred for medical applications [7]. The physicochemical properties of such polymers are better documented, while prediction of degradation mechanisms and lower costs of production are benefits. The most widely used synthetic polymer is poly(lactic acid) (PLA), as approved for medical use by the US Food and Drug Administration (the FDA) [8]. The advantages of PLA include biocompatibility, degradation without toxic by-products, and inexpensive production [8,9]. Materials fabricated solely from PLA have certain limitations, however, including stiffness and a hydrophobic character, hindering their usage [10]. To counteract these issues, supplementation with additives is an option. For example, preparing a PLA material supplemented with PVA improves the flexibility of materials intended for wound healing [11,12,13]. Combining PLA with PVA also enhances the hydrophilicity and ductility of the resulting product [10,14]. Another finding is that PVA acts as an emulsifier when preparing materials from PLA and hydrophilic modifiers, e.g., hydrophilic drugs (W/O emulsion system) [15].

As for the wound-healing process, infected wounds are characterized by the formation of exudate that can create an ideal microenvironment for bacteria to colonize in, thereby inhibiting the healing process. Fabricating materials characterized by a high capacity for absorption is a way of overcoming this issue. In this context, dressings with a porous structure permit access to oxygen (maintaining hydration and the regular distribution of cells), and ensure adequate wettability and hydrophilicity (improving the adhesion of cells to polymer matrices in a hydrolytic biological environment and reducing the chance of biofilm formation by bacterial adhesion) [10,16,17].

Modifying wound dressings with antimicrobial drugs is another means of reducing the risk of infection and heightening the efficiency of the healing process [18,19]. Such wound dressings go beyond the provision of mechanically protecting against infected tissue by affording targeted treatment with high antibiotic concentrations of a drug being applied topically, eliminating the need for systemic dosing. They can also help when dysfunctional blood vessels in the wound bed impede delivery of medication to the healing tissue when a drug is administered systemically.

Various bacteria exist that give rise to infections of wound and soft tissue (e.g., nosocomial infections occur through the action of particular bacteria). Aminoglycoside antibiotics comprise a group of therapeutic agents, which are antimicrobial drugs for treating a wide range of infections (caused by Gram-negative and Gram-positive bacterial strains), including pathogens which are multidrug-resistant [20,21], one example being amikacin (AMI). AMI is applicable for sophisticated therapy and in the treatment of infections with different etiologies, such as those brought about by *Klebsiella pneumoniaeas*, *Escherichia coli*, *Pseudomonas aeruginosa*, *Staphylococcus epidermidis*, and *Staphylococcus aureus* [22]. AMI is used to treat sepsis and infections of soft tissue and the skin, as well as intra-abdominal infections and those of the joints and bones [22,23,24]. AMI is also used in therapy when another AG proves ineffective.

Some polymeric materials have been reported in the literature that were modified with aminoglycosides for medicinal use, primarily limited to orthopedic matters, e.g., PLA-PLGA bone implants [25], or bone fillers consisting of a polycaprolactone-calcium carbonate/calcium phosphate composite [26]. Research has been conducted on materials for wound healing, although findings pertained to aminoglycosides other than AMI [27,28,29,30]. A few materials modified with AMI for wound healing have appeared in published papers; however, these concerned fabricated materials originating from natural polymers, e.g., chitosan-based porous matrices [31], alginate-based hydrogel membranes [32,33] and collagen [34]. In a previous work by the authors [15], a novel PLA-based material (microparticles) with encapsulated AMI was proposed. A description was given of the difficulties encountered when preparing the AMI-modified polymeric materials founded on hydrophobic synthetic polymers, in this case PLA [15,34]. In brief, the main issue related to the hydrophilicity of AMI, therein exceeding that of other aminoglycosides employed in medication (gentamicin, tobramycin, and especially neomycin).

This study reports on a new design of PLA-based porous materials supplemented with AMI intended for healing wounds and soft tissues. Fabricated by spraying a surface with a liquid [14], the porous matrices (PM) consisted of PLA supplemented with PVA as an emulsifier agent. Porous matrices with and without functional additives were proposed. The physicochemical properties of AMI informed the functional modifiers applied: (i) nanosilica—so as to increase the load of AMI and prolong its release from the matrices, as a consequence of the high affinity of AMI for polar silica [35,36]; and (ii) chitosan—due to its biocompatibility and proven wound healing properties [37]. The characteristics of the fabricated materials are detailed herein, including the release kinetics of AMI, a transdermal absorption test, and morphological and elemental studies. Discussion is also given on the importance of the modifiers applied.

## 2. Results and Discussion

The porous matrices (PM) were fabricated from high-molecular, commercially available PLA 4060D (Mw = 191,000 g/mol [38,39]). The procedure for such fabrication involved spraying the PLA 4060D solution in DCM (S2) onto the aqueous solution (S1), the latter containing PVA and AMI; see Materials and Methods section for details. The PLA concentration of the S2 solution was established as 2% *w*/*v*; production of the material proved inefficient at a lower value, while exceeding it resulted in the apparatus lines becoming clogged, since the density of the solution was too high.

**Figure 1:**
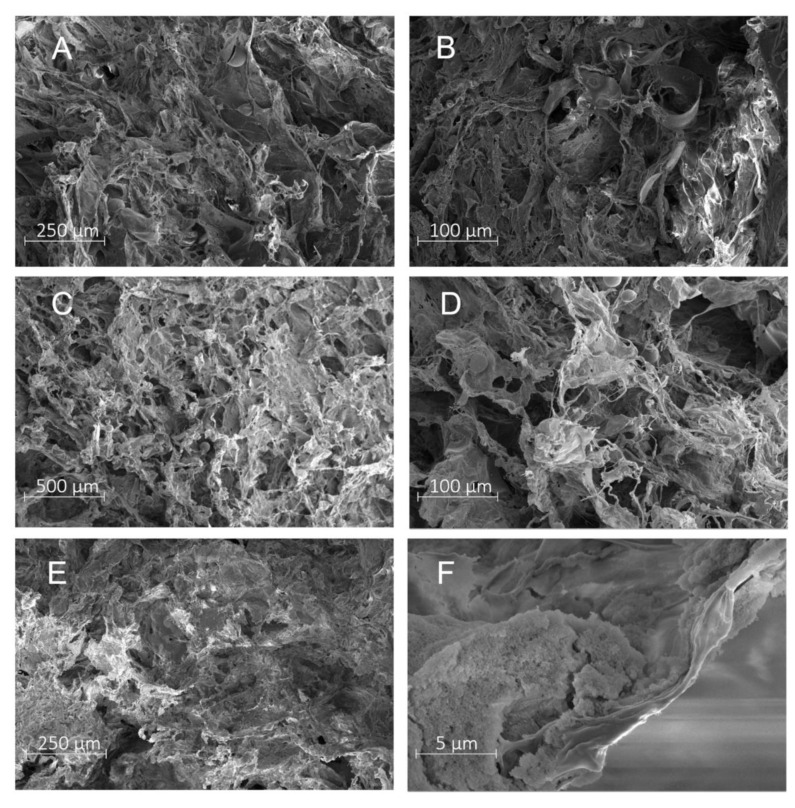
SEM images of the porous matrices; (**A**,**B**) PLA/PVA-AMI; (**C**,**D**) PLA/PVA-CH-AMI; and (**E**,**F**) PLA/PVA-SiO_2_-AMI.

As for the composition of the S1 solution, it had been observed in a previous study that adding PVA was indispensable for obtaining a material with the intended structure. PVA acts as a surfactant, thereby facilitating a dispersed system absent of PLA aggregates in the aqueous medium. Combining PLA with PVA also improved the properties of the resulting product, as the hydrophilicity and ductility of it was greater [10,14]; for this reason, the S1 aqueous solution contained 0.1% *w*/*v* PVA. Figure 1A,B show SEM images of the PLA/PVA-AMI porous matrices; the materials were 5 mm thick and 8 cm in diameter.

Further investigation included the possibility of employing chitosan (CH) to fabricate PM. A notable organic polysaccharide and derivative of chitin, it is widely used in cosmetology (skincare) and the pharmaceutical industry for its biodegradability, non-toxicity, proven antibacterial properties, and its proven wound healing properties [40]. An important application of chitosan is enhancing the treatment of infections. An additional attribute is that chitosan possesses greater flexibility than other natural polymers [41].

Herein, chitosan was used as the sprayed-on component (S2), instead of PLA 4060D. The composition of the aqueous solution (S1) remained the same as before (AMI in 0.1% *w*/*v* PVA solution). Since chitosan is soluble at low pH and completely insoluble under alkaline conditions, a difference in pH was leveraged to produce the material in solid form. The sprayed-on solution consisted of 2% *w*/*v* chitosan in acetic acid (pH = 2), while the acceptor solution comprised 100 mL of 0.5% *w*/*v* AMI and 900 mL H_2_O with the level of pH adjusted to 8 by NaOH. However, spraying did not bring about the solid form as anticipated. A potential reason was that the pH value dropped during the spraying operation onto the aqueous acceptor solution (with the pH of below 6), thereby prompting the chitosan to become soluble [42]. As a consequence, another experiment was carried out with 2% *w*/*v* PLA 4060D in DCM as the S2 solution. As previously mentioned, adding PVA improves the properties of PLA-based materials; hence, the S1 aqueous solution was supplemented with both PVA and CH. The subsequent PLA/PVA-CH-AMI porous matrices possessed a homogeneous structure. An interesting finding was that an organoleptic test revealed the PM modified with chitosan showed immense softness and were delicate to the touch. In contrast, the S1 solution without PVA in combination with 0.1% *w*/*v* chitosan in acetic acid caused stiff, slightly yellow aggregates to form, brought about by the absence of compatibility between the CH and PLA particles [43]. This indicated the solid portion of the PLA adhered to the chitosan and dispersion was poor. The experiment proved that sponge-like materials for wound healing could be prepared from both components (PVA and chitosan), increasing the overall surface area of the material and permitting red blood cells to fully penetrate the pores; in the case of pure chitosan, these stuck to the surface of the chitosan, leading to rapid aggregation on the material itself [44]. The composition of the S1 aqueous solution ultimately consisted of 0.05% *w*/*v* chitosan and 0.05% *w*/*v* PVA in 2% *v*/*v* acetic acid (Figure 1C,D). The thickness of the resultant materials, i.e., 3 mm, was slightly less than that of PM PLA/PVA, while their diameter was equal to 8 cm.

The prepared materials, as indicated earlier, were characterized by slight differences in thickness. This can be clearly described by the difference in the composition of the PM and by the method of PM production (divide the material by weight in Petri dish; see the experimental section and PM fabrication). As a consequence, the highest thickness was noted for PM with silica addition (6 mm). Based on the dry mass of the components, the mentioned PM were fabricated using 1 g of PLA and additionally of 0.8 g of silica (amount of PVA was omitted due to water solubility). The PM composed of only PLA and PVA were characterized by slightly lower thickness (5 mm) due to the lack of additional components. The last type of PM, namely PLA/PVA-CH, was characterized by the lowest thickness (3 mm). Contrary to the previously described PM, the difference in thickness is caused by exposure of the material to acidic conditions (including material preparation and stabilization—almost 24 h), which may cause slight hydrolysis of PLA lower mass of the product [45]. Furthermore, the differences in PM thickness can also result from the different pore volumes for the individual PM (see further—BET analysis results). Porous materials with the same masses always show the increase of the volume with the increase of the porosity. PLA/PVA-SiO_2_-AMI shows the highest total pore volume (0.7900 cm^3^/g), so it also has the highest thickness, which is equal to 6mm. The PM with the lowest thickness (3 mm), which is PLA/PVA-CH-AMI, is characterized by the lowest total pore volume (0.0010 cm^3^/g).

The study then addressed the possibility of supplementing such PLA/PVA-based porous matrices with silica nanoparticles. The beneficial properties of silica, such as biocompatibility, nontoxicity, high specific surface area, and the ability to adsorb active molecules, mark it out as a promising compound for modifying materials intended for drug delivery [35]. The SiO_2_ nanoparticles with diameter of 7 nm (CAS S5130) were selected for this study due to superior dispersion in the aqueous solution, in comparison to spherical ones with the diameter of 200 nm (CAS 748161). The intention was to obtain a material loaded with AMI with a high specific area for wounds that could produce exudation, which would take advantage of the high porosity of the silica [46]. The sprayed solution (S2) consisted of 2% *w*/*v* PLA 4060D in DCM. To formulate the acceptor solution (S1), it was first necessary to modify the silica nanoparticles, whereby the suspension of SiO_2_ in the AMI aqueous solution was prepared and sonicated. This suspension was then added into the PVA solution. Finally, the prepared porous matrices with modified silica were characterized by a homogeneous structure that was 6 mm thick and measured 8 cm in diameter. Figure 1E,F contain SEM images of some samples, the one at higher magnification (Figure 1F) detailing the change in structure compared to the rest of the fabricated PM. Such a change proved the presence of the SiO_2_ nanoparticles on the surface of the material once the PM had been fabricated.

Considering the possible site of drug introduction and binding method, it should be noted that PLA-based materials are characterized by a lack of chemical affinity for the highly polar AMI. Thus, the idea behind the fabrication of the materials was to “trap” the AMI molecules in the structure of the polymer matrix while forming the solid by the method of spraying a surface liquid. To increase the AMI load, the modification of PM with silica nanoparticles was investigated. Considering the properties of silica and AMI, it was concluded that AMI particles probably bind to silica through hydrogen bonding (bonding between silanol groups and AMI amide moieties) [47]. In some cases, like for alumina-silica components, Lewis base (AMI) and Lewis acid (silica) interactions pertained to the related molecular connections. This led to the decision to employ mesoporous nanosilica nanoparticles as a reservoir for the AMI, in accordance with other studies [36,46].

**Figure 2:**
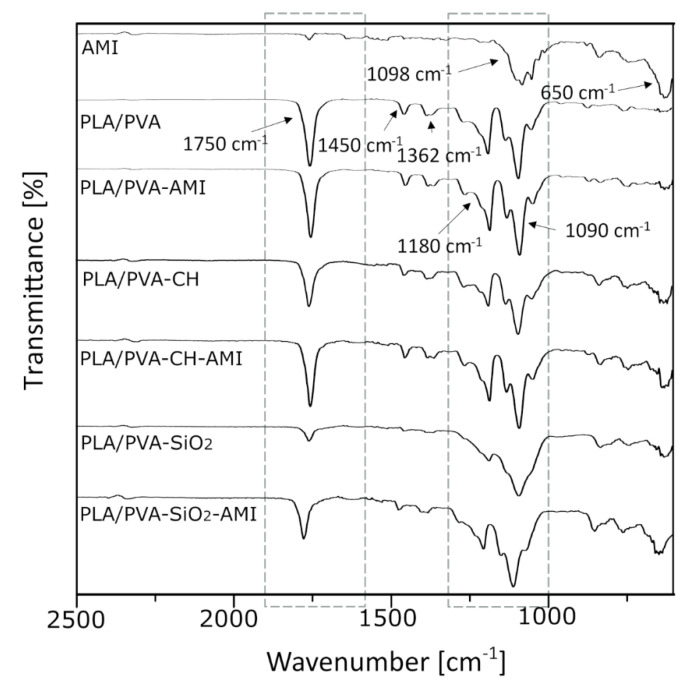
FTIR spectra for the PM before and after modification with AMI.

**Figure 3:**
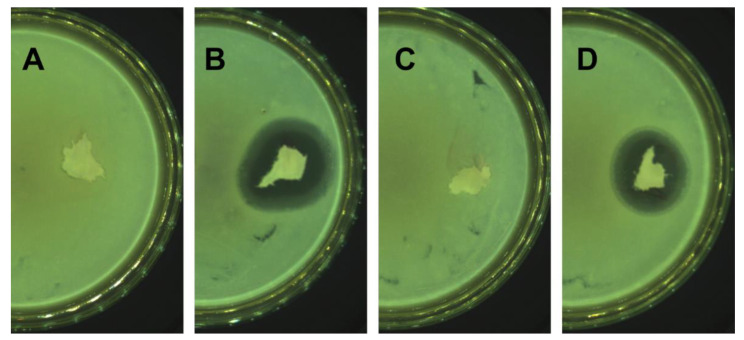
Examples of inhibition zones (against *E. coli*) for (**A**) PM PLA/PVA-CH, (**B**) PM PLA/PVA-CH-AMI, (**C**) PM PLA/PVA-SiO_2_, and (**D**) PM PLA/PVA-SiO_2_-AMI.

These structural alterations prior to and following PM modification were analyzed by FTIR. The outcomes could not be validated as positive, though, as the differences in spectra recorded before and after modification with AMI were minor, a consequence of peaks overlapping for the AMI and components of the porous matrices. With the spectra for AMI sulfate standard in mind (Figure 2), the peak at 1098 cm^−1^ represents SO_4_^2−^ asymmetric stretching vibrations, the absorption band in the region from 1200 to 1000 cm^−1^ is characteristic of C-O stretching bands for carbohydrates, and the peak at ca. 650 cm^−1^ indicates N-H single bond stretching vibrations [48,49]. When comparing the spectra for unmodified PM with the standard for AMI sulfate, the absorption band at 1090 cm^−1^ clearly corresponds to C-O stretching, contributing to the peaks for alcohols masked by the AMI [50]. Other signals relevant to the polymers applied herein in PM fabrication are 1750 cm^−1^ for the C = O carbonyl group, and 1360 cm^−1^ and 1450 cm^−1^ for −CH_3_ (symmetric and asymmetric, respectively) [51]. For materials with silica nanoparticles, the wide-ranging peak at 1090–1010 cm^−1^ pertains to –Si–O–Si bonds [52]. The most characteristic regions are marked with a dotted line (Figure 3).

Based on the EDXRF results (Table 1), it was confirmed that the PM with silica nanoparticles comprised 50–60% *w*/*w* SiO_2_, proving that silica was present in the structure of the fabricated materials. The determined value for silica content was about 10% *w*/*w* lower than the theoretical value; this was recalculated based on the dry mass of the materials upon their preparation, in relation to the 800 mg nanoparticles used for modification. The differences observed could have arisen from incomplete entrapment of the SiO_2_ nanoparticles in the matrix. As for the content of other elements, the presence of 1% *w*/*w* Mg was determined in the PM PLA/PVA and AMI samples. The amounts of elements such as Ca, K, and Cl in all the analyzed samples were negligible, being below the limit of detection (<0.5% *w*/*w*).

The results of Arc Flash analysis (Table 2) revealed that the content of nitrogen in the PM materials increased after loading with AMI, confirming the presence of AMI molecules in the modified materials. The greatest increase in the mass fraction of nitrogen occurred in the porous PM matrices containing SiO_2_, this being ca. 0.6% *w*/*w* higher than for the unloaded PM PLA/PVA-SiO_2_ sample. This had been anticipated by the authors because of the strong affinity polar AMI has to silica nanoparticles.

In relation to the surface areas of the materials, it was found that the SiO_2_ nanoparticles increased the surface area and total pore volume of the PM, brought about by the highly porous SiO_2_ nanoparticles on the surfaces of the PM, in turn creating an irregular structure and enhancing the specific surface of the materials [46,53]. In comparison to the PM without any additive (PM PLA/PVA-AMI), the surface area and total pore volume determined were ca. 3-fold greater (Table 3). The surface area of conventional mesoporous silica has been established as being approximately 1100 m^2^/g [46], although the value obtained for the PM modified with SiO_2_-AMI was less. This reduction in pore size substantiated the loading of the drug, and was related to the PLA/PVA matrix effect, i.e., mean porosity and blocked pores [54]. As previously mentioned, the PLA/PVA-CH matrices supplemented with chitosan were delicate and soft in structure, since they had the smallest pore diameter (8.8 nm), and the sample with chitosan possessed the smallest surface area. A possible explanation for this is that the structure of the PM with chitosan was strongly affected by the type of drug loaded [55]. Hence, the process of pore formation was related to aggregation of the drug and CH particles once the material was freeze-dried during the preparation stage.

The PM were analyzed for antimicrobial activity (Table 4, Figure 3). Since the intended purpose of the PM was to treat the skin and soft tissues, the bacterial strains chosen comprised *S. aureus*, *E. coli*, *E. faecalis*, *P. aeruginosa*, and *K. pneumoniae*. Interestingly, no activity was demonstrated against *E. faecalis* by the PM samples, in contrast to AMI (in the form of an active substance) and SiO_2_-AMI (as applied in PM PLA/PVA-SiO_2_-AMI). This finding verified changes brought about in the microbial action of AMI by the PLA/PVA matrices. Slightly diminished antimicrobial activity was discerned for PM PLA/PVA-SiO_2_-AMI, especially against *E. coli* and *P. aeruginosa*. Similar results were observed after comparing pure AMI and SiO_2_-AMI. It was found that the width of the inhibition zones for materials containing SiO_2_ were lesser in size, potentially as a consequence of the strong interaction between the SiO_2_ and AMI, and the variance in antimicrobial action.

Subsequent studies concerned the determination of the amount of antibiotics in the developed materials (expressed as loading and encapsulation efficiency, % LE and % EE, respectively). Referring to the scientific literature, there are a plethora of PLA-based porous materials described for use as wound dressings. Some of them are loaded with antibiotics to increase therapeutic effects. Examples include: polylactic acid/cellulose acetate-based mats loaded with thymoquinone with % LE 80–91% [56] or PLA nanofibers with encapsulated doxycycline and % LE 5–30% (depending on nanofibers diameters) [57]. Regarding the porous matrices loaded with aminoglycosides, they mainly relate to the use of gentamicin. For example, the PLA/PVA-based materials obtained using also surface solvent spraying method [14]. However, in the mentioned example, the % EE and % LE are presented only as percentage data, without any information on the content of gentamicin taken for matrix preparation. Another investigation was performed on the possibility of fabricating PLA-based porous systems composed of nanofibers. In this case, the possibility of loading different aminoglycosides including AMI were tested [58]. However, finally, only the materials with gentamicin were characterized. Another example is the poly(ε-caprolactone) porous matrices with the % LE as 7% [59]. It is worth noting that there are no similar materials described, and information in the scientific literature in terms of AMI loading, which indicates difficulties in obtaining them.

When considering the developed materials in this study, the highest values of % LE were noted for PM PLA/PVA-AMI and PM PLA/PVA-SiO_2_-AMI (ca. 9%, Table 5). A remarkable aspect was that loading efficiencies determined in terms of AMI in mg per 1 g of polymer were determined as: 96 ± 34 mg/g for PLA/PVA-AMI, 93 ± 13 mg/g for PLA/PVA-SiO_2_-AMI and 25.4 ± 2.2 mg/g for PLA/PVA-CH-AMI. In terms of encapsulation efficiency (% EE), the highest value was achieved for PM PLA/PVA-SiO_2_-AMI (ca. 18%, Table 5), while the lowest for encapsulation and loading efficiency were obtained for PM PLA/PVA-CH-AMI (% EE = 3.11 ± 0.50% and % LE = 2.54 ± 0.22%). This may have happened during the modification stage with chitosan, where some AMI remained in the aqueous solution (the acidic pH of S1 promoted dissolution of the chitosan); hence, the limited portion was essentially integrated with the solid form of PLA. Arc Flash elemental analysis supported this hypothesis (Table 2), since PM PLA/PVA-CH-AMI showed the lowest rise in nitrogen content upon modification at only 0.07% *w*/*w*, compared to the other PM(s). However, excellent antimicrobial efficiency was still observed despite such low levels of % EE and % LE (Table 4).

**Figure 4:**
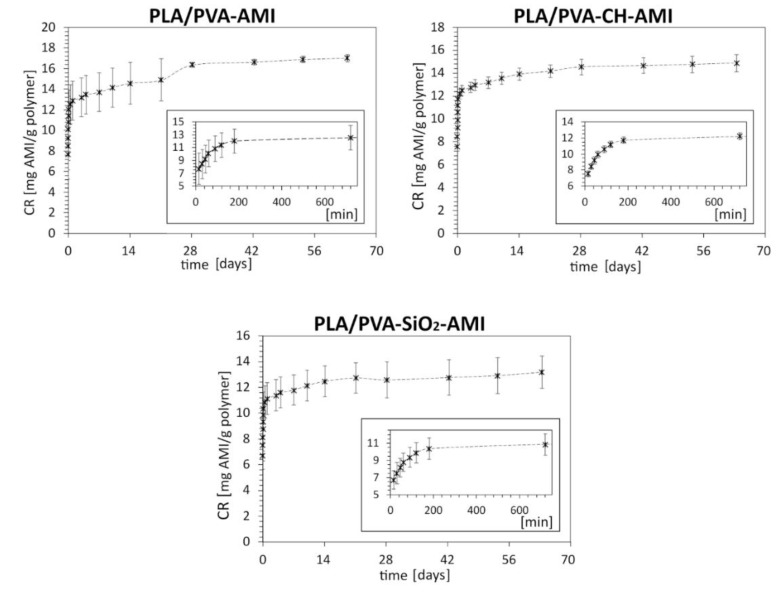
Amikacin release from the PLA/PVA, PLA/PVA-CH, and PLA/PVA-SiO_2_ porous matrices (*n* = 3); the smaller plots show the initial phase of the release process.

According to the in vitro AMI release study (Figure 4, Table S2), the total amount of AMI [in mg] released from the polymer matrix [in g] after 63 days was, respectively, CR = 17.02 ± 0.36 mg/g (% CR = 14.15 ± 0.30% *w*/*w*) for PM PLA/PVA-AMI, CR = 14.88 ± 0.75 mg/g (% CR 62.5 ± 3.2% *w*/*w*) for PM PLA/PVA-CH-AMI, and CR = 13.2 ± 1.3 mg/g (% CR = 8.3 ± 0.79% *w*/*w*) for PM PLA/PVA-SiO_2_-AMI (CR, Equation (3), and % CR, Equation (4)). The lowest CR value was recorded for PLA/PVA-SiO_2_-AMI, despite having the highest % LE and % EE, which could have been caused by the strong affinity of AMI for SiO_2_ nanoparticles, thereby hindering release to the given environment at a pH of 7.4 (PBS solution). The amounts of AMI released after the first 15 min for PM PLA/PVA-AMI and PM PLA/PVA-CH-AMI were at a similar level (respectively, 7.7 ± 2.4 mg/g and 7.56 ± 0.35 mg/g). A slightly lower quantity was gauged for PLA/PVA-SiO_2_-AMI, at 6.7 ± 1.1 mg/g (% CR = 4.22 ± 0.67% *w*/*w*). The extremely rapid release of AMI in the first few minutes of the study related to the presence of the drug, especially on the surface of the PM(s) [60,61].

Table 6 details the constants for the determined AMI release. Based on the R^2^ values, every PM matched best with the Korsmeyer–Peppas model. The values for the diffusion coefficients of all the materials (*n*) were below 0.45 (see Section 3.3.8), proving that the mechanism of AMI release adhered to Fickian diffusion (the most rapid release of AMI was noted during the first 3 h).

**Figure 5:**
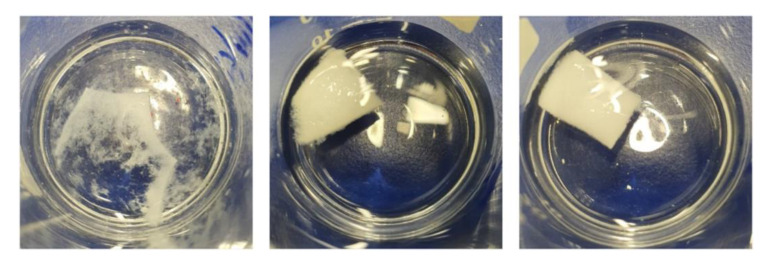
Porous matrices after immersion in PBS solution: PLA/PVA after 5 min (**left**), PLA/PVA-SiO_2_ (**middle**) after 1 h, and PLA/PVA-CH (**right**) after 1 h.

An interesting finding was that, depending on the composition of the porous matrices, different patterns of behavior were observed in structural degradation and stability during exposure to the hydrolytic environment (Figure 5). The PLA/PVA-AMI porous matrices demonstrated the fastest decomposition in PBS solution, after just 5 min of immersion, although the PM samples containing silica and chitosan remained stable even after an hour, without any visible change in structure. However, rapid degradation of PLA/PVA-AMI did not decrease drug release over time. Furthermore, even after a few days, an increase in the concentration of AMI was observed in the release studies. This may be due to the fact that the degradation of the material took place via a macroscopic approach, first causing amikacin to be “ejected” from the pores of the material after the “structure loosening”. From a microscopic point of view, in the bite, fragments of the matrix were present in locations where no damage to the material had occurred, and disintegrated over time.

Figure S2 compares the various materials after 24 h of immersion in PBS at 37 °C as part of the in vitro release test. No further visible degradation of the PLA/PVA-CH-AMI porous matrices took place after this period. For PLA/PVA-Si-AMI after the 30th day of immersion, only single fragments of material (on the order of 3 mm) were observed. Macroscopic decomposition of the structure of the incubated material was not observed. In addition to visual assessment, the samples were analyzed to determine their loss of mass during the immersion experiment (Table 7). In this context, 10 mg of each sample was placed in a flask with 10 mL of PBS solution for 1 h; for PM PLA/PVA-AMI the time was reduced to 15 min, since it decomposed so rapidly. PM PLA/PVA-CH-AMI was characterized by the smallest loss of mass (7.3% *w*/*w*), while the largest was seen for PM PLA/PVA-AMI (30.9% *w*/*w*); the latter occurred as it had the greatest proportion of water-soluble PVA.

According to the degree of swelling determined (Table 7), which corresponded to sorption capacity, all the materials fabricated were characterized by impressive results. The material without additives exhibited the lowest value for swelling ratio, whereas the one supplemented with silica showed ca. 2-fold higher. This phenomenon was brought about by the porosity of the sample and the capacity of silica for water sorption [54]. Such sorption was maximal for the material with chitosan, exceeding PM PLA/PVA-AMI by ca. 4-fold. This was discerned for the PM(s) consisting of chitosan and PVA, for which sorption capacity surpassed the other chitosan-based porous matrices [44,62], this being a consequence of the porosity of the material and swelling by the chitosan polysaccharides [63]. These results supported the notion that the porous matrices were applicable for wounds with high exudation, especially those supplemented with chitosan and silica.

The change in molar masses of the materials after incubation in PBS for 56 days was then considered (Table 8). The initial molecular weight of commercially available PLA 4060D was 191,000 g/mol [38,39]. It was observed that PLA/PVA-AMI and PM PLA/PVA-SiO_2_-AMI possessed slightly lower molecular weight immediately after fabrication (20% less, ca. 150,000 g/mol). In the case of PLA/PVA-CH-AMI, molecular weight equaled merely 3339 g/mol; a potential consequence of the hydrolytic degradation of PLA in the acidified conditions of the fabrication stage, as required for chitosan to be soluble [39]. After 56 days of incubation in the PBS solution, polymer fractions were present in the collected samples, indicating degradation of the materials.

Based on thermogravimetric data, the thermal stability of fabricated materials was characterized. It is worth noting that PM PLA/PVA-SiO_2_-AMI showed the greatest thermal stability, with the highest temperatures being discerned for it, at which 10% and 50% losses in the mass of the sample were recorded. All data and a brief description of the TGA results are included in the Supplementary Materials (see: Figure S3, Table S3 and ‘Comment: TGA analysis—brief discussion’ [64,65,66,67]).

**Figure 6:**
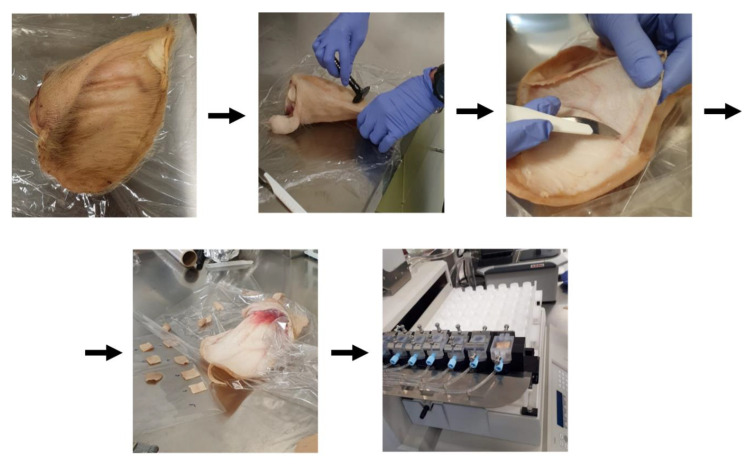
Generalized illustration of the test for transdermal absorption.

Findings relating to antimicrobial analysis and the release kinetics of AMI were satisfactory enough for a simulation of treatment of an infection to be performed with the fabricated porous matrices. The test was carried out in accordance with an OECD-approved method for measuring transdermal drug absorption (Figure 6).

**Figure 7:**
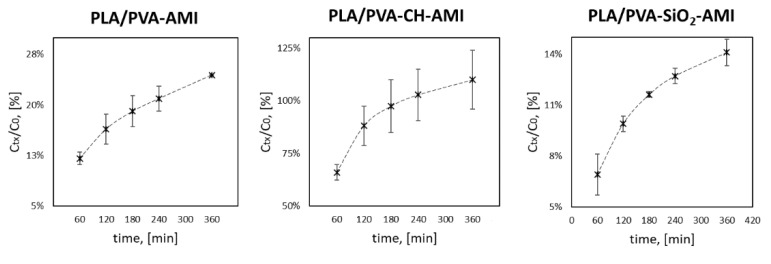
Simulation of AMI release according to OECD guidelines (*C_tx_*—amount of AMI released after time t; *C*_0_—total amount of AMI in the material tested, *n* = 3).

With the highly hydrophilic character of AMI in mind, as well as its inability to be absorbed into deeper skin structures (in contrast to commercially available medicinal formulations such as ointments or creams, where paraffin-based additives, for example, are used to enhance transdermal transport of drug), measurement was carried out as to the antiseptic properties of AMI [68]. An in vitro simulation was performed to this end, consisting of placing the prepared skin (from the ear of a pig) in contact with the fabricated materials modified with AMI in a flow cell. PBS solution was then passed through the system, and the fractions obtained were collected and analyzed by high-performance liquid chromatography to determine the amount of AMI washed out from the materials (*C_tx_*) (Table S2). Figure 7 contains plots for change in AMI over time. As AMI was released in the first few hours, as revealed by the study of release kinetics, analysis occurred for 6 h.

The quantities of AMI (*C_tx_*/*C*_0_) after the first hour of testing were as follows: 12.07 ± 0.92% for PM PLA/PVA-AMI, 66.0 ± 3.7% for PM PLA/PVA-CH-AMI, and 6.9 ± 1.2% for PM PLA/PVA-SiO_2_-AMI. After 4 h of simulation, 100% of AMI had been released from PM PLA/PVA-CH-AMI, while the remainder of the materials exhibited 24.427 ± 0.073% for PM PLA/PVA-AMI and 14.12 ± 0.78% for PM PLA/PVA-SiO_2_-AMI after six hours. In summary, all of the prepared materials were capable of the gradual release of AMI.

## 3. Materials and Methods

### 3.1. Materials

The following were used in the experiments: PLA 4060D (Ingeo, Minnetonka, MN, USA), PVA 80% hydrolyzed (Mw = 9000 ÷ 10,000 g/mol) (Merck, Darmstadt, Germany), amikacin (AMI) disulfate (Interquim, Cuautitlán Izcalli, México), deionized water, chitosan (CH) (low molecular weight, 50,000–190,000 Da) (Sigma Aldrich, Burlington, MA, USA), silica nanoparticles (particle size 7 nm) (Sigma Aldrich, Burlington, MA, USA), dichloromethane (DCM), tetrahydrofuran (THF) stabilized with BHT (Merck), acetonitrile (ACN), ammonium acetate (Sigma Aldrich, Burlington, MA, USA), o-phthaldialdehyde (OPA), acetic and boric acid (Sigma Aldrich, Burlington, MA, USA), NaOH, KOH, NaCl, KCl, dipotassium phosphate (Lach-Ner, Neratovice, Czech Republic), monosodium dihydrogen orthophosphate (PENTA, Prague, Czech Republic), and gentamicin sulfate (Sigma Aldrich, Burlington, MA, USA); polystyrene standards 580–6,000,000 g/mol (Polymer Laboratories Ltd., Church Stretton, UK). The bacterial strains (Tomas Bata University in Zlin, Czech Republic) tested against were: *Staphylococcus aureus* CCM 4516, *Escherichia coli* CCM 4517, *Enterococcus faecalis* CCM 3956, *Klebsiella pneumoniae* CCM 4415 and *Pseudomonas aeruginosa* CM 1961.

Phosphate-buffered saline (PBS) was prepared by dissolving: 0.2 g of KCl, 8 g of NaCl, 1.44 g of Na_2_HPO_4_, and 0.24 g of KH_2_PO_4_ in 1 L of distilled water. The pH value of the buffer was adjusted to 7.4 using NaOH.

### 3.2. Fabrication of the Porous Matrices

**Figure 8:**
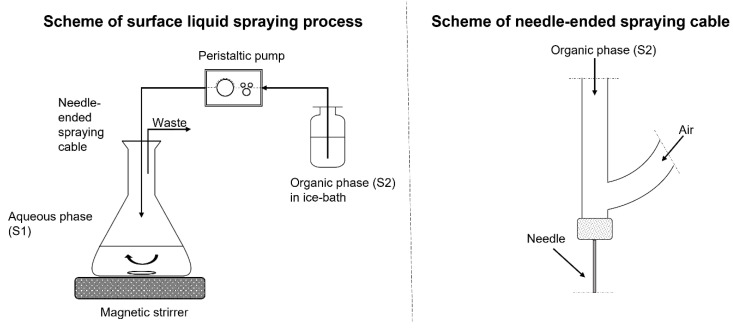
Instrumental setup for PM preparation.

In Figure 8, the scheme of PM fabrication is presented. Table 9 summarizes the compositions of the aqueous and organic solutions used to produce the PM under the study.

#### 3.2.1. Preparation of PLA/PVA-AMI Porous Matrices

A bottle with 2% *w*/*v* PLA 4060D solution in DCM (S2) was placed in an ice bath. Next, the PLA solution was sprayed over the surface of aqueous solution (S1) with the help of compressed air stream (1 bar), through a cable terminated with a needle (I.D. 0.6 mm). The flow rate of the sprayed solution was 0.2 mL/min. Once spraying was finished, the resulting suspension was mixed overnight (300 rpm) in order to remove any residual DCM. The suspension was then vacuum filtered, and the precipitate divided into 3 equal parts (by mass). Aliquots of precipitate were placed in Petri dishes (I.D. 8 cm) together with 20 mL of deionized water. Precipitate was evenly distributed over the surface of Petri dishes with the help of gentle vortexing and mixing with a glass rod. The resultant material was frozen and lyophilized.

#### 3.2.2. Preparation of the PLA/PVA-CH-AMI Porous Matrices

The procedure for preparation of the PLA/PVA-CH-AMI porous matrices was identical to that described earlier, the only difference was the composition of S1 solution.

#### 3.2.3. Preparation of the PLA/PVA-SiO_2_-AMI Porous Matrices

In the case of the preparation of PLA/PVA-SiO_2_-AMI porous matrices, the same procedure as above was followed. The aqueous solution (S1) was prepared in the following way: 800 mg of Si nanoparticles (7 nm) was added to 100 mL of 0.5% *w*/*v* AMI solution, and the suspension was then sonicated for 30 min, after which it was combined with 900 mL of PVA (1 g) and mixed (600 rpm) for 24 h.

### 3.3. Characterization Methods

#### 3.3.1. Scanning Electron Microscopy (SEM)

The AMI-modified and reference (unmodified) porous matrices were analyzed on a scanning electron microscope, a Nova NanoSEM 450 (5 kV) unit. The samples were sputtered with a layer of gold and palladium (SC7620 Mini Sputter Coater Quorum Technologies, Laughton, England).

#### 3.3.2. Fourier Transform Infrared Spectroscopy—Attenuated Total Reflectance (FTIR-ATR)

FTIR-ATR analysis was carried out on a Nicolet iS5 FTIR spectrometer fitted with an iD5 ATR accessory (resolution: 4 cm^−1^, 64 scans, optical material: Ge).

#### 3.3.3. Elemental Analysis

Elemental studies (CHO, Ca, S, Cl, Mg, K, and Si) were conducted by means of X-ray fluorescence (EDXRF) on a Thermo Scientific ARL Quant X device set to the He mode of measurement; the FLASH method was employed as a complementary test to determine the content of C, H, N, O, and S; the presence of N_2_, CO_2_, H_2_O, and SO_2_ were gauged by GC-TCD after Dumas combustion.

#### 3.3.4. Brunauer–Emmett–Teller (BET) Surface Area Analysis

The samples were degassed for 3 h (40 °C) and analyzed according to the Micrometrics BET method for surfaces (Belsorp-mini II, BEL Japan, Inc., Osaka, Japan).

#### 3.3.5. Thermogravimetric Analysis (TGA)

A TGA Q500 device (TA Instruments, New Castle, DE, USA) was employed under an N2 atmosphere for such analysis. The heating program was set to 10 °C/min; the range in temperature was 25 °C–500 °C (for the porous matrices without silica), and 25 °C–900 °C (for the porous matrices supplemented with silica).

#### 3.3.6. Microbiological Properties—Disk Diffusion Method (Kirby–Bauer Method)

Samples of the modified and unmodified porous matrices (identical in mass) were incubated at 35 °C (18–24 h) on inoculated Mueller–Hinton agar. The width of zones were measured to the nearest millimeter. Antibacterial activity was investigated through the application of five bacterial strains (10^6^–10^7^ cfu/mL): *Escherichia coli* CCM 4517, *Staphylococcus aureus* CCM 4516, Klebsiella pneumoniae CCM 4415, *Pseudomonas aeruginosa* CM 1961, and *Enterococcus faecalis* CCM 3956.

#### 3.3.7. High-Performance Liquid Chromatography (HPLC)

Determination of AMI took place through in-needle derivatization with an o-phthaldialdehyde (OPA) reagent and FLD detection (HPLC Dionex UltiMate 3000 Series, Thermo Fisher Scientific, Waltham, MA, USA).

A Waters XSELECT CSH C18 (4.6 × 250 mm, 5 µm) column fitted with a SecurityGuard column (Phenomenex) was employed. The temperature of separation was established at 30 °C, the process transpiring under isocratic mode (55:45 *v*/*v*) with 100 mM acetate buffer (pH 5.8) as eluent A and ACN as eluent B; the flow rate was 0.4 mL/min; FLD detection was carried out at λ_excitation_ = 330 nm and λ_emission_ = 440 nm. The in-needle derivatization adhered to a specific injection program, as follows: 1 µL original sample solution, 5 μL borate buffer (5 g H_3_BO_3_/100 mL, pH = 11 with KOH), 3 μL OPA reagent*, and 1 μL 1 M acetic acid.

* OPA reagent: 2.5 mg OPA + 400 µL MeOH + 200 µL reducing solution** + 4.4 mL borate buffer.

** Reducing solution: 250 µL 2-mercaptoethanol + 10 mL buffer solution (I).

The stock solution of AMI (1 mg/mL) was diluted with PBS solution (pH = 7.4) to prepare standard solutions (0.5–100 µg/mL). The external calibration method was applied to determine the amount of AMI. Limits of detection and quantification were established as LOD = 0.19 μg/mL and LOQ = 0.58 μg/mL, respectively. The calibration curve obtained was linear (R^2^ = 0.9994) within the analyzed ranges of concentration.

#### 3.3.8. In Vitro Release Study in Liquid Media

Fifty milligrams of the porous matrices were immersed in 10 mL of PBS (pH 7.4) and then incubated at 37 °C (with gentle shaking, at 100 rpm). At the specified time, 1 mL of liquid fraction was collected and replaced with 1 mL of fresh PBS solution. The porous matrices were prepared in triplicate and analyzed 3 times by HPLC.

Encapsulation efficiency (EE, Equation (1)) and loading efficiency (LE, Equation (2)) were determined as follows:

(1) $$\%EE=\frac{C_{t}-C_{f}}{C_{t}}$$

(2) $$\%LE=\frac{C_{t}-C_{f}}{W_{m}}$$

where *C_t_* is the total concentration of AMI applied for modification, *C_f_* represents the concentration of “free” AMI in the solution after fabricating the porous matrices, and *W_m_* stands for the dry mass of the porous matrices.

Before determining the profile for AMI release, values for cumulative release (CR) and cumulative release in per cent (%CR) were calculated (Equations (3) and (4), respectively):

(3) $$CR=\frac{CR_{t}}{M}$$

where *CR_t_* is the amount of AMI (in mg) released at the determined time of *t*, and *M* represents the mass of the porous matrices [g];

(4) $$\%CR=\frac{CR_{t}}{C_{0}}\cdot 100$$

where *C*_0_ is the amount of loaded AMI [mg].

The release kinetics of AMI were investigated using different mathematical models, namely zero-order kinetics (Equation (5)), first-order kinetics (Equation (6)), the Higuchi equation (Equation (7)) and the Korsmeyer–Peppas model (Equation (8)):

(5) $$\frac{CR_{t}}{CR_{fin}}=Kt$$

(6) $$\frac{CR_{t}}{CR_{fin}}=1-e^{-Kt}$$

(7) $$\frac{CR_{t}}{CR_{fin}}=Kt^{\frac{1}{2}}$$

(8) $$\frac{CR_{t}}{CR_{fin}}=Kt^{n}$$

where *CR_fin_* is the total amount of AMI released [mg], K denotes constant release, and *n* constitutes the diffusion coefficient (*n* ≤ 0.45 represents Fickian diffusion; 0.45 < *n* < 1 is characteristic for anomalous transport [69]).

#### 3.3.9. Gel Permeation Chromatography (GPC)

GPC was carried out to determine the mass degradation of materials after 56 days of exposition in PBS solution. A 1 mL volume of the liquid phase of the sample was collected (samples were incubated in the same manner as in tests for the in vitro release described below). The liquid fraction was then evaporated, the solid residue re-dissolved by THF, and the product subsequently filtrated and analyzed.

GPC conditions: set of connected LC columns: PL gel of types MIXED-A (300 × 7.8 mm, 20 µm), MIXED-B (300 × 7.8 mm, 10 µm), and MIXED-D (300 × 7.8 mm, 5 µm) and THF as the mobile phase; the flow rate of the mobile phase was 1 mL/min; the temperature of LC column 40 °C.

A refractive index detector (RID) and viscometric detector (VISC) were employed for detection purposes. Calibration adhered to the polystyrene standards of 580–6,000,000 g/mol; the injection volume was 100 μL.

#### 3.3.10. Transdermal Absorption—In Vitro Study

Transdermal tests were carried out in accordance with the “OECD Test Guidelines for Skin Absorption: The In Vitro Method”. The initial stage involved preparing receptor fluid (2 L), which consisted of PBS buffer and 0.05% *w*/*v* gentamicin sulfate. The test subject was skin from the ear of a pig. The area inside the ear lobe was shaved, and the inner portion of the auricle was separated from the underlying cartilage using a scalpel. Square skin samples with dimensions of 3 × 3 cm were cut out from the separated skin. The skin samples were then placed in test cells and their integrity was determined on a UNI-T UT71D multimeter; samples with a resistance of below 5 mΩ were excluded.

Samples of individual materials were placed on the prepared skin samples. Testing was performed on an automated diffusion cell system with continuous flow of the receptor liquid through the samples (Fraction Collector FC33, Permegear, Northampton, PA, USA). The receptor fluid was collected for 24 h at a constant flow rate of 2 mL/h.

After measurements had been taken, the fractions obtained at preset times (1–6 h) were stored at −20 °C prior to analysis, in preparation for which the samples were filtrated and analyzed by liquid chromatography (HPLC-FLD, under the conditions described above).

## 4. Conclusions

Novel porous and biodegradable PLA-based materials for healing wounds and soft tissues were proposed in this study. To obtain the necessary antimicrobial properties, the PLA porous matrices were initially modified with a polar aminoglycoside antibiotic—amikacin.

All the prepared materials exhibited antimicrobial properties against the bacterial strains of *S. aureus*, *E. coli*, *P. aeruginosa*, and *K. pneumoniae*, and shared release kinetics for AMI that followed the Korsmeyer–Peppas mathematical model, i.e., the fastest release of AMI was observed in the first hours of treatment, followed by a slower rate of release during following days.

The PM samples varied as a result of the functional additives applied, characterized by the different initial amounts of AMI released, and in their porosity, thereby permitting adjustment of the material depending on the desired therapeutic effect.

The first-studied porous matrices composed of PLA/PVA were characterized by rapid macro-scale structure decomposition in a hydrolytic environment. However, even after decomposition of the matrix at the macro scale, the AMI trapped in the PLA structural fragments was still released in a prolonged manner, over several dozen days. Taking into account this behavior, the biocompatibility of the materials used in their manufacture, and the proven antimicrobial properties of AMI, the suggested use for this material would be as a “soluble” material for treating infections of internal organs or skin.

The possibility of using mesoporous silica and chitosan as functional additives was also studied. It was found that adding silica and chitosan improved the stability of the PM(s) in a hydrolytic environment. The PM samples with chitosan were characterized by the most delicate structure to the touch (which would raise patient comfort during treatment), while also demonstrating an impressive liquid sorption capacity as well as the highest structural stability in the hydrolytic environment. This material seems to be well suited to the treatment of skin infections with high exudation. PM with the addition of silica showed a slightly lower stability in the hydrolytic environment and weaker antimicrobial properties compared to PM with chitosan. In fact, they could be used in a similar way to chitosan PM, but for minor infections with less exudate.

## Figures and Tables

**Table 1:** Results of the elemental analysis (EDXRF) of the materials in the form of porous matrices.

Sample	CHON [%] *	Si [%]	S [%]	Mg [%]
PM PLA/PVA	98.80	-	-	0.87
PM PLA/PVA-AMI	97.80	-	-	-
PM PLA/PVA-CH	96.70	-	-	-
PM PLA/PVA-CH-AMI	97.60	-	-	-
PM PLA/PVA-SiO_2_	49.30	49.84	-	-
PM PLA/PVA-SiO_2_-AMI	41.10	58.47	-	-
AMI	86.40	-	12.60	1.00

* weight in percent.

**Table 2:** Results of elemental analysis (Arc Flash) of the materials in the form of porous matrices.

Sample	N ± SD * [%] **	C ± SD [%]	H ± SD [%]	S ± SD [%]
PM PLA/PVA	0.65 ± 0.49	50.802 ± 0.054	5.54 ± 0.13	-
PM PLA/PVA-AMI	0.999 ± 0.055	50.767 ± 0.051	6.037 ± 0.023	-
PM PLA/PVA-CH	0.496 ± 0.040	50.82 ± 0.11	5.995 ± 0.064	-
PM PLA/PVA-CH-AMI	0.57 ± 0.33	49.991 ± 0.029	5.816 ± 0.055	-
PM PLA/PVA-SiO_2_	0.53 ± 0.15	37.65 ± 0.39	4.590 ± 0.055	-
PM PLA/PVA-SiO_2_-AMI	1.13 ± 0.12	29.78 ± 0.53	3.721 ± 0.039	-
AMI	8.114 ± 0.010	30.18 ± 0.22	6.364 ± 0.072	11.5 ± 1.2

* SD—three independently prepared material samples, analyzed twice; ** weight in percent.

**Table 3:** Results of BET analysis for PM.

Sample	Surface Area [m^2^/g]	Mean Pore Diameter [nm]	Total Pore Volume (*p*/*p*_0_ = 0.990) [cm^3^/g]
PLA/PVA-AMI	32.987	33.20	0.2743
PLA/PVA-CH-AMI	0.6280	8.80	0.0010
PLA/PVA-SiO_2_-AMI	94.362	33.50	0.7900

**Table 4:** Antibacterial activity of fabricated porous matrices.

Sample	Width of the Inhibition Zone ± SD [mm]				
	*S. aureus*	*E. coli*	*E. faecalis*	*P. aeruginosa*	*K. pneumoniae*
PLA/PVA	0.0	0.0	0.0	0.0	0.0
PLA/PVA-AMI	7.0 ± 0.0	7.0 ± 0.0	0.0	6.5 ± 0.5	9.5 ± 0.5
PLA/PVA-CH	0.0	0.0	0.0	0.0	0.0
PLA/PVA-CH-AMI	8.5 ± 0.5	8.0 ± 0.0	0.0	6.5 ± 0.5	7.5 ± 0.5
PLA/PVA-SiO_2_	0.0	0.0	0.0	0.0	0.0
PLA/PVA-SiO_2_-AMI	7.5 ± 0.5	5.5 ± 0.5	0.0	4.5 ± 0.5	6.5 ± 0.5
AMI-SiO_2_	13.0 ± 0.0	12.5 ± 0.5	15.0 ± 0.0	14.0 ± 0.0	12.5 ± 0.5
AMI	16.5 ± 0.5	16.5 ± 0.5	11.0 ± 0.0	19.0 ± 0.0	18.0 ± 0.0

SD—three independently prepared material samples, analyzed in triplicate.

**Table 5:** Encapsulation and loading efficiency of the porous matrices modified with AMI.

Sample	% EE ± SD * [%]	% LE ± SD [%]
PM PLA/PVA-AMI	13.6 ± 4.2	9.6 ± 3.4
PM PLA/PVA-CH-AMI	3.11 ± 0.50	2.54 ± 0.22
PM PLA/PVA-SiO_2_-AMI	18.2 ± 1.3	9.3 ± 1.3

* SD—three independently prepared material samples, analyzed in triplicate.

**Table 6:** Release constants (*K*) and correlation coefficients (R^2^) determined by mathematical models.

	0-Order Release		First-Order Release		Higuchi Equation		Korsmeyer–Peppas Model	
Sample	*K*_0_ ± SD *	R^2^	*K_I_* ± SD	R^2^	*K_H_* ± SD	R^2^	*K_KP_* ± SD	R^2^
PM PLA/PVA-AMI	0.296 ± 0.015	0.8000	0.48 ± 0.14	0.8891	0.285 ± 0.042	0.9029	0.522 ± 0.032	0.9791
PM PLA/PVA-CH-AMI	0.3122 ± 0.0020	0.7015	0.5171 ± 0.0050	0.9411	0.3013 ± 0.0014	0.8336	0.5855 ± 0.0040	0.9958
PM PLA/PVA-SiO_2_-AMI	0.298 ± 0.015	0.6768	0.48 ± 0.38	0.9509	0.289 ± 0.013	0.8162	0.551 ± 0.033	0.9977

* SD—three independently prepared material samples, analyzed in triplicate.

**Table 7:** Comparison of the loss of mass and sorption capacities of the porous matrices during immersion.

Sample	Loss of Mass of the Material ** [% *w*/*w*]	Loss of PBS Solution [% *v*/*v*]	Swelling Ratio *** [% *w*/*w*]
PM PLA/PVA-AMI *	30.9	7.0	1779.7
PM PLA/PVA-CH-AMI	7.3	11.0	6602.4
PM PLA/PVA-SiO_2_-AMI	8.2	9.0	3466.6

* Time of immersion: 15 min (PM PLA/PVA-AMI) and 1 h (PM PLA/PVA-CH-AMI and PM PLA/PVA-SiO_2_-AMI); ** loss of mass calculated according to the mass of the sample taken for testing and the dry mass of sample after immersion; *** ratio of mass of the PM with water (directly after immersion) to the dry mass of the material.

**Table 8:** GPC results after 0 and 56 days of degradation in PBS solution.

Sample	Mw [g/mol]	Mn [g/mol]	Mp [g/mol]	Đ [–]
PM PLA/PVA-AMI (0 days)	150,350	105,590	146,125	1.42
PM PLA/PVA-AMI (56 days)	1678	1289	1008	1.30
PM PLA/PVA-CH-AMI (0 days)	3339	2798	2645	1.19
PM PLA/PVA-CH-AMI (56 days)	2526	1496	810	1.69
PM PLA/PVA-SiO_2_-AMI (0 days)	148,730	120,258	129,298	1.24
PM PLA/PVA-SiO_2_-AMI (56 days)	1219	937	796	1.30

Mw—weight average molar mass; Mn—number average molar mass, Mp—molar mass at the maximum of peak; Đ—molar mass dispersity (Đ = Mw/Mn).

**Table 9:** Composition of the fabricated material, in brief.

Acronym	Aqueous Solution Composition (S1)	Organic Solution Composition (S2)
PLA/PVA-AMI	0.05% *w*/*v* AMI with 0.1% *w*/*v* PVA in water	2% *w*/*v* PLA 4060D in DCM
PLA/PVA-CH-AMI	0.05% *w*/*v* AMI with 0.05% *w*/*v* PVA and 0.05% *w*/*v* chitosan in 2% *v*/*v* acetic acid aqueous solution	2% *w*/*v* PLA 4060D in DCM
PLA/PVA-SiO_2_-AMI	0.05% *w*/*v* AMI with 0.08% *w*/*v* SiO_2_ and 0.1% *w*/*v* PVA	2% *w*/*v* PLA 4060D in DCM

## Data Availability

Not applicable.

## Supplementary Materials

The following supporting information can be downloaded at: https://www.mdpi.com/article/10.3390/molecules27207045/s1, Figure S1: Example of fabricated PM material (PLA/PVA). Figure S2: Porous matrices after 24 h of immersion in PBS (pH 7.4), 37 °C (during “in-vitro” release testing): PLA/PVA-AMI (left), PLA/PVA-SiO_2_-AMI (middle), PLA/PVA-CH-AMI (right). Table S1: AMI release form PLA/PVA, PLA/PVA-CH and PLA/PVA-SiO_2_ porous matrices. CR and % CR determined using Equations (3) and (4). Table S2: Results of simulation of AMI release according to OECD guidelines. Figure S3. Thermogravimetry and differential thermogravimetry curves for the porous matrices of PLA/PVA-AMI (A), PLA/PVA-CH-AMI (B), and PLA/PVA-SiO_2_-AMI (C) and amikacin (D). Table S3: Results of TGA analysis for porous matrices (peak numbers according to Figure S3). Comment: TGA analysis—brief discussion.
