# Supplementary material for: Antibacterial Porous Systems Based on Polylactide Loaded with Amikacin
Source: Molecules. 2022 Oct 19;27(20):7045. doi: 10.3390/molecules27207045 (PMC9609933; doi:10.3390/molecules27207045)
Supplement: Supplementary file 1 [file molecules-27-07045-s001.zip › molecules-1943823-supplementary.pdf]

Supplementary

Antibacterial Porous Systems based on Polylactide loaded with Amikacin

Marta Glinka[1], Katerina Filatova[2], Justyna Kucińska-Lipka[3], Tomáš Šopík[2], Eva Domincová Bergerová[2], Veronika Mikulcová[2], Andrzej Wasik[1,*], Vladimir Sedlařík[2]

[1] Department of Analytical Chemistry, Faculty of Chemistry, Gdańsk University of Technology, 11/12 G. Narutowicza Street, 80-233 Gdańsk, Poland
[2] Centre of Polymer Systems, University Institute, Tomas Bata University in Zlín, Tomáše Bati 5678 Street, 760-01 Zlín, Czech Republic
[3] Department of Polymer Technology, Faculty of Chemistry, Gdańsk University of Technology, 11/12 G. Narutowicza Street, 80-233 Gdańsk, Poland

* Correspondence: wasia@pg.edu.pl

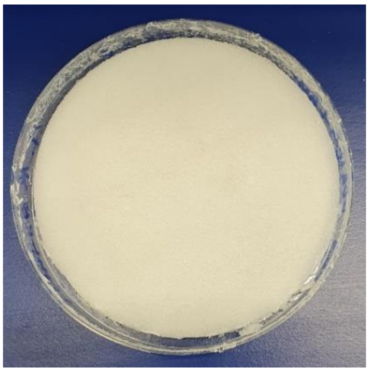

Figure S1. Example of fabricated PM material (PLA/PVA).

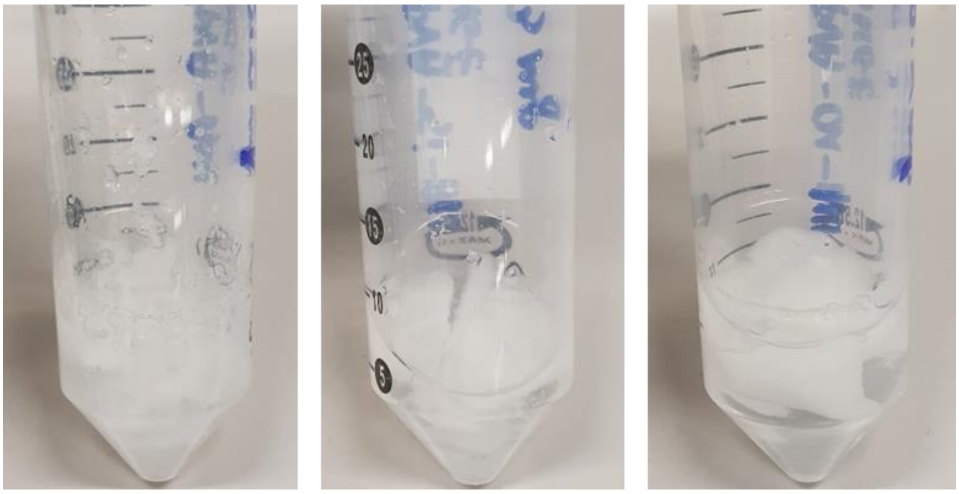

Figure S2. Porous matrices after 24 h of immersion in PBS (pH 7.4), 37°C (during "*in-vitro*" release testing): PLA/PVA-AMI (left), PLA/PVA-SiO_2-AMI (middle), PLA/PVA-CH-AMI (right).

Table S1. AMI release form PLA/PVA, PLA/PVA-CH and PLA/PVA-SiO_2 porous matrices. CR and %CR determined using Eq. (3) and (4).

	PM PLA/PVA-AMI			PM PLA/PVA-CH-AMI			PM PLA/PVA-SiO_2-AMI		
Time, [min]	%CR ± SD*,[%]	CR ± SD,[mg AMI/g PM]	CV,[%]	%CR ± SD*,[%]	CR ± SD,[mg AMI/g PM]	CV,[%]	%CR ± SD*,[%]	CR ± SD,[mg AMI/g PM]	CV,[%]
15	6.4 ± 2.0	7.7 ± 2.4	31.8	31.8 ± 1.5	7.56 ± 0.35	4.6	4.22 ± 0.67	6.7 ± 1.1	15.8
30	7.0 ± 1.9	8.5 ± 2.3	26.8	35.5 ± 1.4	8.45 ± 0.34	4.0	4.74 ± 0.78	7.5 ± 1.2	16.5
45	7.7 ± 1.8	9.2 ± 2.1	23.2	38.7 ± 1.8	9.21 ± 0.44	4.7	5.13 ± 0.70	8.2 ± 1.1	13.5
60 (1 h)	8.4 ± 1.7	10.1 ± 2.1	20.8	41.7 ± 1.8	9.93 ± 0.43	4.4	5.53 ± 0.69	8.8 ± 1.1	12.4
90 (1.5 h)	9.0 ± 1.7	10.8 ± 2.0	18.4	44.5 ± 1.8	10.59 ± 0.42	4.0	5.89 ± 0.72	9.4 ± 1.1	12.3
120 (2 h)	9.5 ± 1.6	11.4 ± 1.9	16.7	47.0 ± 1.7	11.20 ± 0.40	3.5	6.23 ± 0.74	9.9 ± 1.2	11.9
180 (3 h)	10.0 ± 1.6	12.0 ± 1.9	15.7	49.2 ± 1.7	11.72 ± 0.40	3.4	6.54 ± 0.78	10.4 ± 1.2	11.9
720 (12 h)	10.4 ± 1.6	12.5 ± 1.9	15.2	51.2 ± 1.8	12.20 ± 0.43	3.6	6.84 ± 0.79	10.9 ± 1.3	11.6
1440 (1 day)	10.7 ± 1.6	12.9 ± 1.9	14.6	52.4 ± 1.9	12.49 ± 0.44	3.6	7.02 ± 0.78	11.1 ± 1.2	11.1
4320 (3 days)	11.0 ± 1.6	13.2 ± 1.9	14.2	53.5 ± 2.0	12.75 ± 0.47	3.7	7.17 ± 0.76	11.4 ± 1.2	10.7
5760 (4 days)	11.2 ± 1.5	13.5 ± 1.9	13.8	54.5 ± 2.0	12.97 ± 0.47	3.7	7.31 ± 0.75	11.6 ± 1.2	10.3
10080 (7 days)	11.4 ± 1.5	13.7 ± 1.9	13.5	55.3 ± 2.1	13.17 ± 0.50	3.8	7.43 ± 0.74	11.8 ± 1.2	9.9
14400 (10 days)	11.8 ± 1.6	14.2 ± 1.9	13.4	56.9 ± 2.2	13.55 ± 0.51	3.8	7.65 ± 0.74	12.1 ± 1.2	9.7
20160 (14 days)	12.1 ± 1.7	14.6 ± 2.0	13.9	58.4 ± 2.2	13.90 ± 0.52	3.8	7.86 ± 0.75	12.5 ± 1.2	9.6
30240 (21 days)	12.4 ± 1.7	14.9 ± 2.1	13.8	59.5 ± 2.3	14.17 ± 0.54	3.8	8.02 ± 0.74	12.7 ± 1.2	9.3
40320 (28 days)	13.59 ± 0.19	16.35 ± 0.28	1.4	61.1 ± 2.9	14.54 ± 0.69	4.7	7.93 ± 0.88	12.6 ± 1.4	11.1
60480 (42 days)	13.83 ± 0.23	16.63 ± 0.28	1.7	61.5 ± 2.9	14.65 ± 0.69	4.7	8.04 ± 0.87	12.8 ± 1.4	10.9
76320 (53 days)	14.04 ± 0.25	16.89 ± 0.30	1.7	62.0 ± 3.0	14.76 ± 0.71	4.8	8.14 ± 0.88	12.9 ± 1.4	10.8
90720 (63 days)	14.15 ± 0.30	17.02 ± 0.36	2.1	62.5 ± 3.2	14.88 ± 0.75	5.1	8.30 ± 0.79	13.2 ± 1.3	9.6

*SD – 3 independently prepared material samples, analyzed in triplicate; CV – variation coefficient

Table S2. Results of simulation of AMI release according to OECD guidelines.

	PM PLA/PVA-AMI		PM PLA/PVA-CH-AMI		PM PLA/PVA-SiO_2-AMI	
Time, [min]	(C_{tx}/C_0) ± SD*, [%]	CV, [%]	(C_{tx}/C_0) ± SD*, [%]	CV, [%]	(C_{tx}/C_0) ± SD*, [%]	CV, [%]
60	12.07 ± 0.92	7.6	66.0 ± 3.7	5.6	6.9 ± 1.2	17.6
120	16.4 ± 2.2	13.7	88.2 ± 9.3	10.6	9.91 ± 0.46	4.6
180	19.0 ± 2.3	12.1	97 ± 13	12.9	11.63 ± 0.16	1.4
240	20.9 ± 1.9	0.9	103 ± 12	11.9	12.73 ± 0.45	3.5
360	24.427 ± 0.073	0.3	110 ± 14	12.8	14.12 ± 0.78	5.5

*SD – samples were prepared twice and analyzed in triplicate

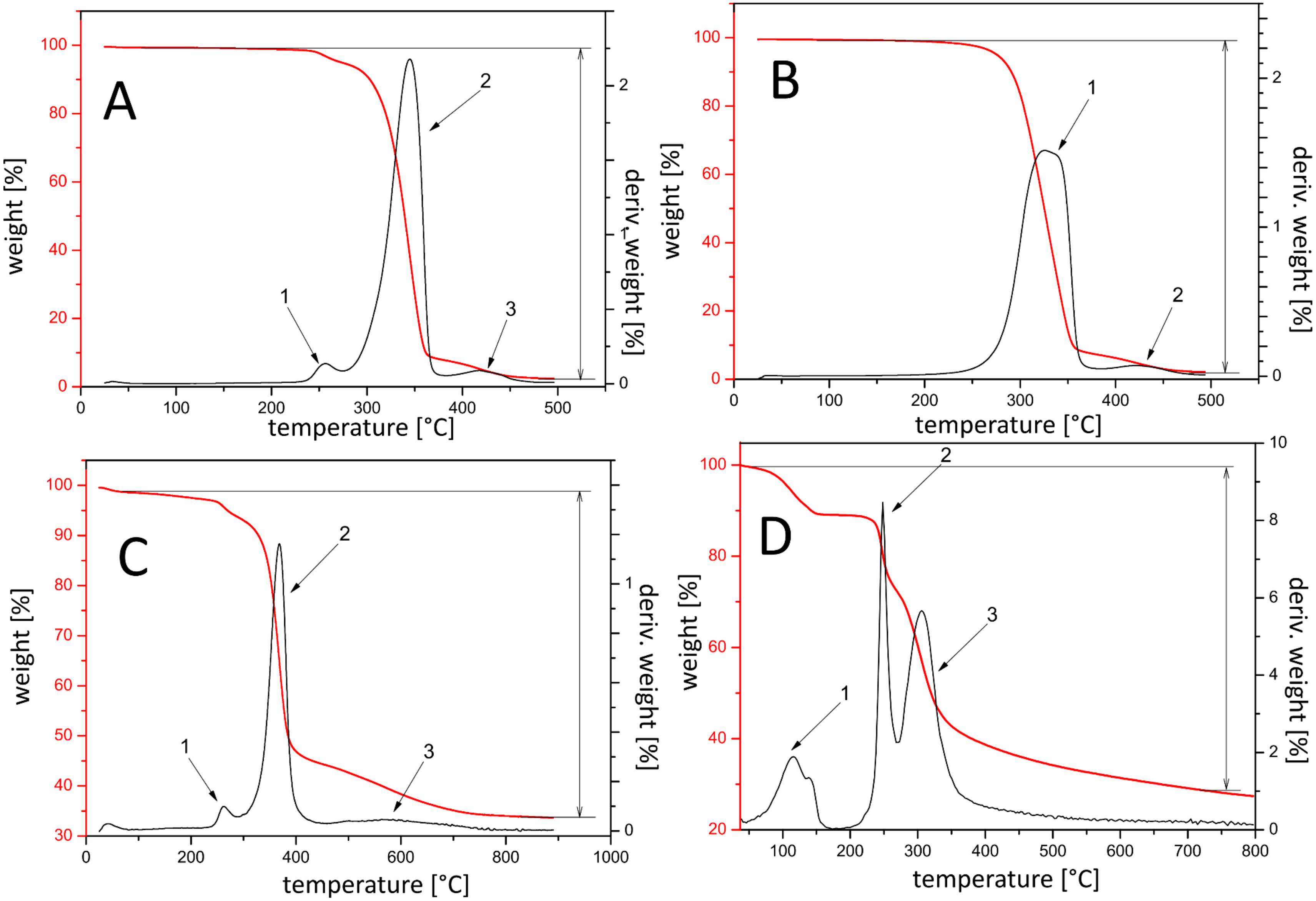

Figure S3. Thermogravimetry and differential thermogravimetry curves for the porous matrices of PLA/PVA-AMI (A), PLA/PVA-CH-AMI (B), and PLA /PVA-SiO_2-AMI (C) and amikacin (D).

Table S3. Results of TGA analysis for porous matrices (peak numbers according to Fig. S3).

	Peak 1				Peak 2				Peak 3				Δw_{fin}	$\Delta T_{5\%}$, [°C]	$\Delta T_{10\%}$, [°C]	$\Delta T_{50\%}$, [°C]
Sample	T_i, [°C]	T_m, [°C]	T_f, [°C]	Δw	T_i, [°C]	T_m, [°C]	T_f, [°C]	Δw	T_i, [°C]	T_m, [°C]	T_f, [°C]	Δw				
PM PLA/PVA-AMI	225	256	277	4.0	277	345	375	86.5	375	424	457	5.3	95.8	273	303	340
PM PLA/PVA-CH-AMI	227	326	363	91.3	363	423	492	6.1	-	-	-	-	97.4	276	291	325
PM PLA/PVA-Si-AMI	243	263	298	4.9	298	368	502	50.6	502	585	817	8.6	64.1	269	328	385
AMI	50	114	170	11.0	200	247	275	14.0	275	302	500	40.0	70.0	104	137	319

T_i – onset temperature; T_m – temperature corresponding to maximum mass loss rate; T_f – final temperature; Δw – mass loss in the range: $T_i \div T_f$; Δw_{fin} – total mass loss; $\Delta T_{5\%}$, $\Delta T_{10\%}$, $\Delta T_{50\%}$ – temperature corresponding to respectively 5%, 10% and 50% of sample mass loss

Comment: TGA analysis – brief discussion

Thermogravimetric analysis (Fig. S3, Table S3) revealed that temperatures corresponding to the maximum rate of loss in mass varied according to the composition of the polymer matrix of the materials; i.e. 345°C for PM PLA/PVA-AMI, 326°C for PM PLA/PVA-CH-AMI, and 368°C for PM PLA/PVA-SiO_2-AMI. In the literature a slight reduction in mass is reported, from 50°C to ca 150°C, primarily caused by the vaporization of moisture, which is especially visible in the case of AMI (as an API, see Figure S3-D) or other volatile products [64-65]. All the materials studied herein demonstrated loss in mass at approximately 300–400°C, indicating the decomposition of PLA [66]. The wide peak at this region for PM PLA/PVA-CH-AMI also showed partial overlapping of another component with a similar temperature of decomposition (200°C to 360°C), this being chitosan and AMI, see Figure S3 [65,67]. PVA decomposed at 400–450°C, brought about by further degradation of polyene [65]. The peak at ca 250°C could denote the decomposition of AMI. Temperatures corresponding to initial degradation of the materials (the onset temperature for peak 1, Fig. S3) were 225°C and 227°C for PM PLA/PVA-AMI and PM PLA/PVA-CH-AMI, respectively. In the case of PM PLA/PVA-AMI, the loss in mass observed during the first stage of measurement (see Fig. S3, peak 1) was 4% w/w, and the highest value was seen for PM PLA/PVA-CH-AMI (90.3% w/w). It was found that the initial onset temperature was the highest (243°C) for PM PLA/PVA-SiO_2-AMI, as a consequence of adding the silica. For PM PLA/PVA-AMI, the first peak indicated loss in mass equal to 4.9% w/w. The lowest such loss was observed for PM PLA/PVA-SiO_2-AMI (50.6% w/w) in comparison to the other materials, caused by the presence of the silica (the melting point of which is above 1400°C). The peak at 585°C for PM PLA/PVA-SiO_2-AMI (Fig. S3, peak 3) suggested partial phase conversion of the silica had taken place (quartz β > 573°C); loss in mass at this temperature was 8.6% w/w. Table S3 summarizes the values of $\Delta T_{5\%}$, $\Delta T_{10\%}$, and $\Delta T_{50\%}$, corresponding to the temperatures at which 5%, 10%, and 50% of loss in mass of the samples had occurred, respectively. The materials were similar in terms of the temperature at which 5% loss in mass happened (269–276°C). It is worthy of note that PM PLA/PVA-SiO_2-AMI showed the greatest thermal stability, and the highest temperatures were discerned for it at which 10% and 50% of loss in mass of the sample were recorded
